# Regional Variations in Growth Plate Chondrocyte Deformation as Predicted By Three-Dimensional Multi-Scale Simulations

**DOI:** 10.1371/journal.pone.0124862

**Published:** 2015-04-17

**Authors:** Jie Gao, Esra Roan, John L. Williams

**Affiliations:** 1 Departments of Mechanical Engineering, University of Memphis Memphis, Tennessee, 38152, United States of America; 2 Department of Biomedical Engineering, University of Memphis Memphis, Tennessee, 38152, United States of America; National University of Ireland Galway, IRELAND

## Abstract

The physis, or growth plate, is a complex disc-shaped cartilage structure that is responsible for longitudinal bone growth. In this study, a multi-scale computational approach was undertaken to better understand how physiological loads are experienced by chondrocytes embedded inside chondrons when subjected to moderate strain under instantaneous compressive loading of the growth plate. Models of representative samples of compressed bone/growth-plate/bone from a 0.67 mm thick 4-month old bovine proximal tibial physis were subjected to a prescribed displacement equal to 20% of the growth plate thickness. At the macroscale level, the applied compressive deformation resulted in an overall compressive strain across the proliferative-hypertrophic zone of 17%. The microscale model predicted that chondrocytes sustained compressive height strains of 12% and 6% in the proliferative and hypertrophic zones, respectively, in the interior regions of the plate. This pattern was reversed within the outer 300 μm region at the free surface where cells were compressed by 10% in the proliferative and 26% in the hypertrophic zones, in agreement with experimental observations. This work provides a new approach to study growth plate behavior under compression and illustrates the need for combining computational and experimental methods to better understand the chondrocyte mechanics in the growth plate cartilage. While the current model is relevant to fast dynamic events, such as heel strike in walking, we believe this approach provides new insight into the mechanical factors that regulate bone growth at the cell level and provides a basis for developing models to help interpret experimental results at varying time scales.

## Introduction

The physis, or growth plate, is a complex disc-shaped cartilage structure that is responsible for longitudinal bone growth. This growth is modulated by many systemic and local factors including those arising from mechanical loading [1–3]. While the underlying mechanisms are still under investigation, several mechanical candidates have been explored as potential tissue level signals for modulating endochondral bone formation, e.g. hydrostatic and octahedral shear stresses and principal stresses [4–9]. However, chondrocytes, are the active agents of growth and contribute to bone growth through cell proliferation, hypertrophy and extracellular matrix secretion in highly specialized, highly cellular and organized anatomic structures known as chondrons. It is likely that tissue level strains and stresses are experienced differently by chondrocytes embedded within such structures, possibly providing signals of varying intensity and type depending on location.

Mechanotransduction is more readily related to cell deformation than to tissue level stresses. In the context of bone growth, there is evidence that cell proliferation and differentiation can be regulated through activation of stretch-activated ion channels in the cell membrane following changes in cellular shape and size [10–13]. Although computational studies incorporating various bone growth theories have been applied at the gross tissue level, to our knowledge none have been reported that relate tissue level mechanics to chondrocytes within an environment that faithfully represents the unique microstructural anatomy of growth plate cartilage.

Recent observational studies of physeal samples under compression have revealed regional variations in cellular strains and suggest that global physeal strains may be amplified at the cellular level [12, 14]. Such observational studies are essential to our understanding of the relation between form, function, mechanical deformation and bone growth regulation. However, current experimental techniques for studying chondrocyte deformation require sectioning through the physis to expose a free surface, thereby altering the very strain distribution of interest and compromising efforts to quantify the cellular response in the physiologic state.

The aim of this study was to develop a multi-scale finite element model of the growth plate to estimate strains experienced by chondrocytes under instantaneous physiologic compression. Specifically, we sought to answer the following two questions: 1) Do chondrons and chondrocytes close to the cutting surface (free surface) experience similar deformations to those located in the central region of the growth plate? 2) What is the relationship between computed chondron- and cell-level strains under compressive loading and the cellular anatomy and physiology of the growth plate?

## Materials and Methods

**Fig 1:**
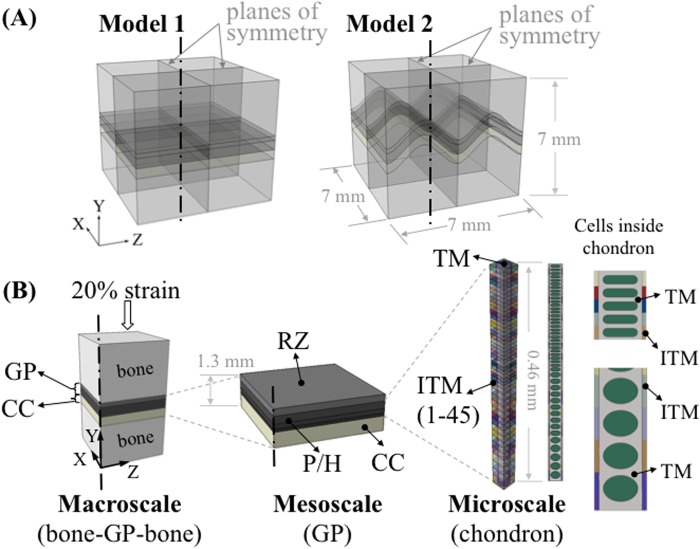
Overview of the multiscale modeling approach. (A) Two idealized models were constructed consisting of epiphyseal bone, growth plate (GP) cartilage and metaphyseal bone (about 7 x 7 x 7 mm) with variations of mammillary processes: flat and ‘m’ shaped. (B) Set-up of the multiscale modeling approach. At the macroscale level, quarter models were used for analysis. About 0.69 mm thick growth plate cartilage was partitioned into two sections to represent the reserve zone (RZ) and the proliferative/hypertrophic (P/H) zone. Calcified cartilage (CC) was also included in the macroscale model. At the mesoscale, three individual layers were generated in the P/H zone to represent the gradient change of elastic modulus through the thickness of the growth plate. The microscale model of the chondron consisted of interterritorial matrix (ITM), territorial matrix (TM) and 46 chondrocytes with gradually changing cellular shape along with the same number of ITM sections. The elastic modulus of ITM increased from the RZ to the metaphyseal side to represent the gradual change of its material properties.

We used a multiscale finite element submodeling approach in this study. Submodeling is useful to study a local region of a model, based on the interpolation of nodal results from its global model onto the nodes at the boundaries of the submodel [15]. This allows us to study the transmission of loads from the macroscopic structure (in millimeters) to microscopic structures (in microns). Our model (Fig 1) is comprised of a macroscale model to represent a uniaxial compression test sample (bone-growth plate-bone), a mesoscale model to represent the growth plate with its various zones, and a microscale model to represent the axial columnar organization of chondrocytes (chondrons) in the proliferative and hypertrophic zones (P/H zone).

We modeled two simplified shapes (flat (Model 1) and an ‘m’ shape (Model 2) with an amplitude of 1.13 mm) of the growth plate layer (Fig 1A) representing different stages of growth. At the early stage of the bone development, e.g. at birth until the appearance of secondary ossification center, the growth plate cartilage has a relatively flat shape [16–18]. As the growth plate cartilage ossification proceeds, mammillary processes appear and the growth plate forms more complex contours [16–18]. Similar growth plate shapes have been used in finite element models in previous studies [19, 20].

Homogeneous isotropic linear elastic materials were used for each component in a large displacement analysis to simulate the response to instantaneous loading along the bone axis (Y-axis). A uniform displacement of 0.134 mm was prescribed on the surface of the epiphyseal bone of the test sample equaling 20% of the 0.67 mm growth plate cartilage thickness. Since only the instantaneous response was considered in this study, the time dependent mechanical responses of each component are not captured and the effect of the cells is reasonably to be ignored for simplicity [21–25]. Material properties were chosen based on published values in the literature (Table 1). Mesh convergence studies were conducted for all models at each scale and the nonlinear geometric effects were taken into account for all models. Reduced integration formulation was used to prevent volumetric locking and save computational time. Hybrid elements with enhanced hourglass control (C3D8RH for macroscale and mesoscale models, C3D4H for microscale model) were utilized on incompressible materials to remedy potential locking problems during the analyses.

**Table 1:** Young’s modulus (E) and Poisson’s ratio (ν) of the growth plate components used in the multi-scale models.

		E (MPa)		ν	
		value	references	value	references
Macroscale	Trabecular bone	100	[20]	0.3	[20]
	Reserve zone	0.98	Adapted	0.47	[24]
	P/H zone	0.49	Adapted	0.47	[24]
	Calcified cartilage	9.68	[26]	0.3	[24]
Mesoscale	Reserve zone	0.98	Adapted	0.47	[24]
	P/H zone 1	0.38		0.47	[24]
	P/H zone 2	0.6		0.47	[24]
	P/H zone 3	0.83		0.47	[24]
Microscale	ITM1	0.42	[26]	0.45	[24]
	ITM45	1.87	[26]	0.45	[24]
	TM	0.26	[27]	0.45	[24]
	Chondrocytes	0.002	[28–32]	0.5	[24, 33]

Values for E in the inter-territorial matrix (ITM) varied between level 1 and 45.

### Macroscale: bone-growth plate-bone

A finite element model from our previous study [34] was utilized with material properties which represent the instantaneous response. Compared to equilibrium state properties, there are fewer published studies for instantaneous state properties [24]. The few available studies indicate that the instantaneous (short time scale) elastic modulus and Poisson’s ratio values are higher than those for the equilibrium state [24, 35, 36]. Based on literature and the intrinsic elastic modulus that was extracted from our previous study on a bovine growth plate [34], 0.62 MPa and 0.47 were assumed as the instantaneous elastic moduli and Poisson’s ratio, respectively.

The growth plate layer was partitioned into two sections to represent the reserve zone (RZ) and the proliferative/hypertrophic (P/H) zone. The proportion of each section was assumed based on previous experimental data [37]. The elastic modulus of the reserve zone was assigned to be twice that of the P/H zone according to previous studies [38, 39] while keeping the composite elastic modulus of the whole growth plate layer at the value stated above. Calcified cartilage was assigned a value of 9.68 MPa for the elastic modulus [26]. This model consisted of 181,056 elements (C3D8R).

### Mesoscale: growth plate (RZ, P/H and calcified cartilage zones)

**Fig 2:**
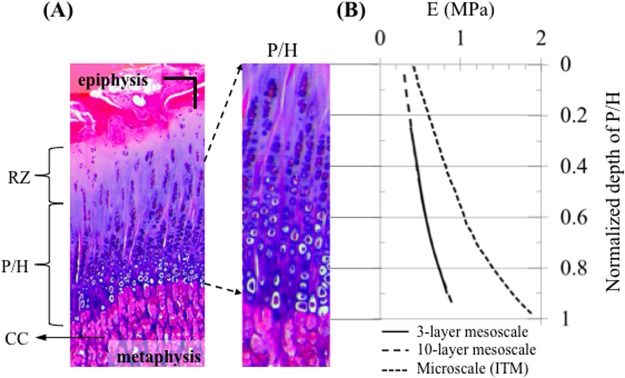
Determination of the elastic moduli of the growth plate utilized in the models. **(A)** Stained histological slice (haematoxylin and eosin) of a 4-month old bovine growth plate; chondrocytes are dispersed in the reserve zone (RZ) and are stacked in tubes (chondrons) extending from the zone of proliferation (P) through the zones of maturation and hypertrophy (H). The walls of the chondron tubes (interterritorial matrix) become increasingly calcified toward the metaphyseal side. Following chondrocyte death, the walls of tubes or bars of calcified cartilage (CC) matrix form the scaffolding upon which bone is laid down (primary spongiosa); scale bar = 100 μm. **(B)** Distribution of the elastic modulus of the P/H zone (3-layer mesoscale and 10-layer mesocale models) and interterritorial matrix (ITM) (microscale model).

Three individual layers were generated in the P/H zone of the growth plate (Fig 1B) to represent the change of elastic modulus through the thickness of the cartilage (Table 1) [36]. The elastic moduli of the individual layers of the P/H zone were adjusted iteratively (Fig 2) so that the overall effective stiffness of the 3-layer mesoscale model was 0.49 MPa, which is the same as the model with a single P/H layer (macro-scale model). An intermediate model (not shown) consisting of 10 layers in the P/Z zone was created to reduce the influence of mismatch of elastic modulus between the 3-layer P/H zone in the mesoscale model and the microscale chondron model containing 45 cells. The same iterative experimental-data-informed method was used to adjust individual layer moduli so that the overall stiffness was the same as that for the macroscale model, i.e. an effective response of 0.49 MPa. In order to obtain the effective response of the 3-layered or 10-layered mesoscale models, each multilayered mesoscale model was uniaxially compressed and a stress-strain response was obtained to compute an effective elastic modulus. This is similar to the inverse methods that we carried out in our previous work in which we determined the intrinsic elastic modulus of the bovine growth plate [34]. The resulting input material parameters of the mesoscale models are shown in Fig 2. Three-layered and 10-layered model consisted of 159,848 and 119,700 C3D8RH elements, respectively.

### Microscale: chondron

This model was built to represent the tubular (“columnar”) structure of a chondron in the P/H zone. It consists of interterritorial matrix (ITM), territorial matrix (TM) and chondrocytes (Fig 1B). Chondrocytes from the proliferative zone were modeled as cells with flat top and bottom surfaces, whilst chondrocytes from hypertrophic zone were modeled as ellipsoids. The heights of the 20 μm wide and deep chondrocytes varied with the location to represent the cellular shape changes along the axial direction of the chondron. For the transition from the proliferative to hypertrophic zone in the axial direction, three more cell shapes were employed to represent the gradual shape change of the chondrocytes. The dimensions of the chondron tube were assumed to be 30 μm X 30 μm (width X depth).

A total of 45 chondron sub regions each consisting of a chondrocyte and a homogeneous ITM were modeled in the microscale model. The elastic modulus of the ITM has been shown to increase from the reserve zone to the hypertrophic zone [26]. Therefore, in the model, the elastic modulus of the ITM increased from 0.42 MPa (region close to the reserve zone) to 1.87 MPa (region close to the calcified cartilage zone). Material properties were initially selected based on previous studies [27] and adjusted iteratively, with the use of unconfined uniaxial compression simulations using a data-driven modeling approach, so that the composite stiffness matched that of the mesoscale model (0.49 MPa) (Fig 2). The elastic modulus of the chondrocytes was assigned a value of 2 kPa based published values for articular cartilage chondrocytes [28–32]. In order to simulate the instantaneous response, the chondrocytes were assumed to be incompressible with Poisson’s ratio of 0.5 [24, 33]. The material properties of the TM and chondrocytes were constant throughout the depth of the chondron. The chondron model consisted of 268,455 C3D4H elements for cells and C3D4 elements for the TM and ITM.

In order to investigate the influence of different transverse locations of the chondron, four to five transverse locations (from the center toward the free surface of the macroscale models) were selected to implement this microscale model for three macroscale models with different growth plate shapes. The microscale model was only implemented to a depth of about 300 μm away from the free surface due to severe mesh distortion at or adjacent to the free surface.

### Implementation

The implicit solver of a commercial finite element program, ABAQUS v6.12 (Simulia, Providence, RI), was used in this study. At the macroscale, quarter models were built by imposing symmetric boundary conditions along YZ and XY planes leaving two free surfaces parallel to the XY and YZ planes with symmetric boundary conditions.

A mesh convergence study was performed at each scale level of this series of finite element models in order to determine an appropriate element size. For the macroscale and mesoscale models we used a convergence criterion of 5% or less change in the maximum value of Von Mises stress. For the microscale model, the average value of Von Mises stress within the first chondrocyte, which is closest to the reserve zone, was used as the criteria for convergence.

Since large deformations occurred at each scale level of the model, the effect of geometric nonlinearity can be significant. Therefore, NLGEOM was turned on throughout the analysis to take this into account. Except where otherwise noted all strain results extracted directly from the analysis are presented as logarithmic strains (LE). Cellular height and width strains were calculated as engineering strain from measurements of distance between flat cell surfaces or using a ‘best-fit ellipsoid’ approach similar to that described in another study [40].

Bone samples shown in images were obtained from Swissland Packing Company (Ashkum, IL), a veal slaughterhouse and meat processing company. The samples were from 18-week old calves.

## Results

**Fig 3:**
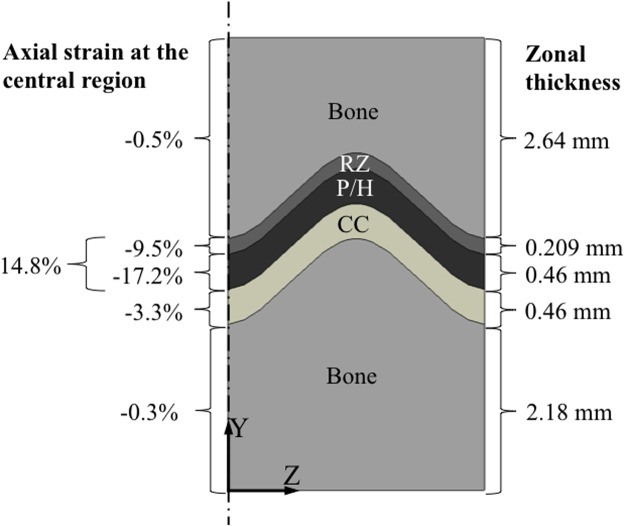
Different levels of axial strain were experienced by different zones (layers) of the sample. Although a compressive displacement equal to 20% of the growth plate thickness was applied to the top surface of the whole sample, the combined reserve zone and proliferative/hypertrophic (P/H) layers were subjected to engineering strains of only about 15%. Strains in the calcified cartilage (CC) zone reached about 3% strain.

The prescribed compressive displacement which was equivalent to 20% of the initial uncompressed combined initial thicknesses (0.67 mm) of RZ and P/H zones resulted in a -14.8% growth plate engineering strain across the combined layers of the reserve, proliferative and hypertrophic zones, while the combined proliferative and hypertrophic zone received -17.2% strain (Fig 3). The reserve zone experienced -9.5% and the calcified cartilage zone -3.3% compressive strain. This strain distribution is dependent on the assumed material properties and relative thicknesses of each zone. The latter are known to vary with age and stage of development of the growth plate. The values used in the model are representative of a 4-month old bovine proximal tibial physis.

**Fig 4:**
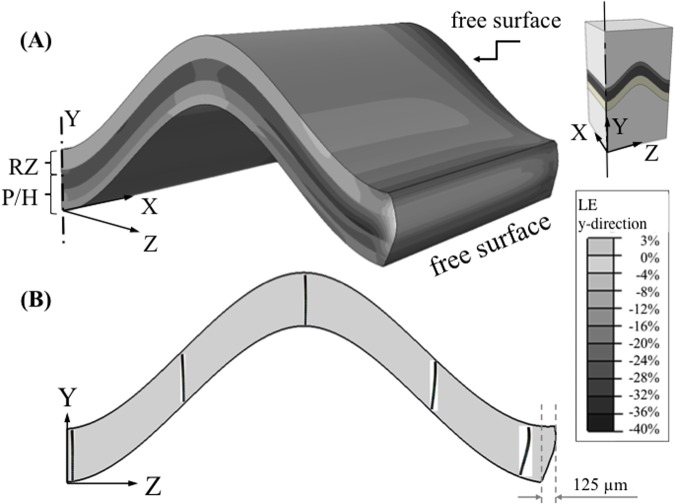
Distribution of the axial logarithmic strain and deformation of the chondrons. (A) A non-uniform distribution of axial logarithmic strain, in the y-direction, exists within the ‘m’ shape growth plate in the mesoscopic model. Chondrocytes were not included in this model. The distribution of strain was significantly different at these locations from the central or internal regions of the growth plate and reached values approaching double the nominal engineering strain of 20%; (B) Deformed chondrons obtained from the microscale model with cellular detail is overlaid on a deformed macroscale model displaying only the growth plate. Chondrons buckled within 300 μm of free surfaces, but not within the growth plate interior when 20% overall compressive strain was applied across the macroscale model. Deformed models are not scaled up. The ‘m’ shape growth plate exhibited transverse outward buckling deformation of 125 μm at the edge. The contour plot is obtained by extrapolating the integration point values to the nodes and then averaging.

The axial (Y-axis) logarithmic strain distribution along the bone long axis direction in the mesoscale model of the growth plate was highly non-uniform and reached peak strains of 40% in the proliferative zone near the free boundary (Fig 4A). Chondrons, the primary functional and structural units of the growth plate, deformed by buckling near the free surfaces where the cartilage bulged outward while remaining straight in the interior regions (Fig 4B). The shape of the mammillary process also influenced the degree of buckling at the free surface. Flat growth plate exhibited greater buckling deformations of chondrons near the periphery where the maximum transverse (outward) displacement was 158 μm as compared with 125 μm for the ‘m’ shape growth plate (flat plate results are not shown).

**Fig 5:**
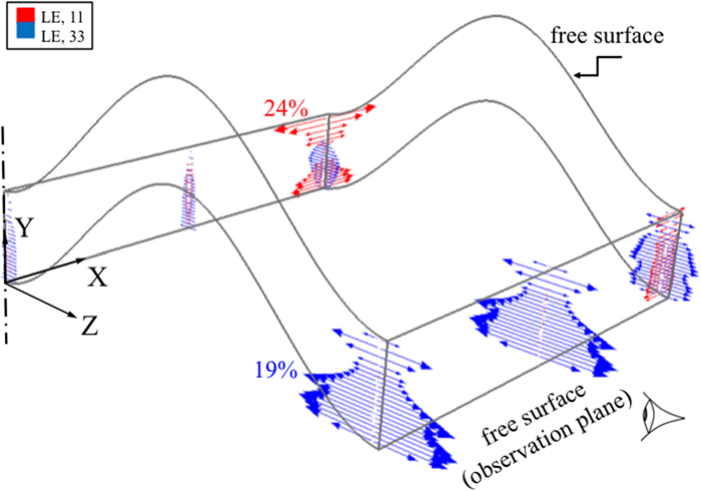
Logarithmic strain vector plots at the center and at a plane 50 μm from one of the two free surfaces of the ‘m’ shape growth plate undergoing axial (Y-) compression of 20%. The red and blue arrows represent strains in X and Z directions, respectively, at the integration points. The results were obtained from the mesoscale model where detailed chondrons are not included. Regions close to the outer edge experienced a significant level of transverse (X- and Z-) strains. These strains suggest that observations made near the surface would lead to different assessment of transverse outward strain distribution compared to the interior of the growth plate. The centerline represents the center of the full model, as a quarter of the actual growth plate layer is shown here (XY and YZ planes are symmetry planes).

In the transverse (X- and Y-axes) directions significant out of plane strains developed (Fig 5) around the exterior surfaces throughout all of the growth plate zones, but especially in the hypertrophic and reserve zones. Transverse strains were at a minimum at the base of the proliferative zone where cell division occurs.

**Fig 6:**
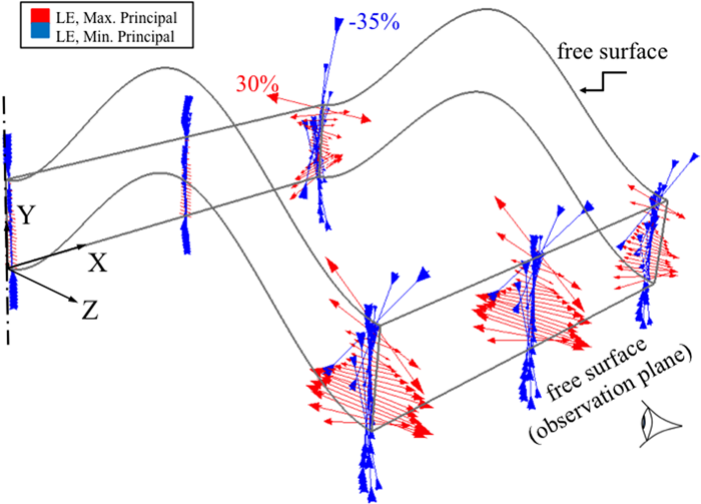
Principal logarithmic strain vector plots at center and at 50 μm from one of the free surfaces of the ‘m’ shape growth plate. The arrows represent the maximum and minimum principal strains. The results were obtained from the mesoscale model without the details of chondrons. Regions close to the outer edges experienced a significant level of ‘out-of-plane’ strain. The free surfaces caused significant changes in the principal strains near the epiphyseal bone border. Tensile strains were greatest at this border and in the hypertropic zone at the calcified cartilage border and smallest at the base of the proliferative zone where cell division takes place. The centerline represents the center of the full model, as a quarter of the actual growth plate layer is shown here (XY and YZ planes are symmetry planes).

**Fig 7:**
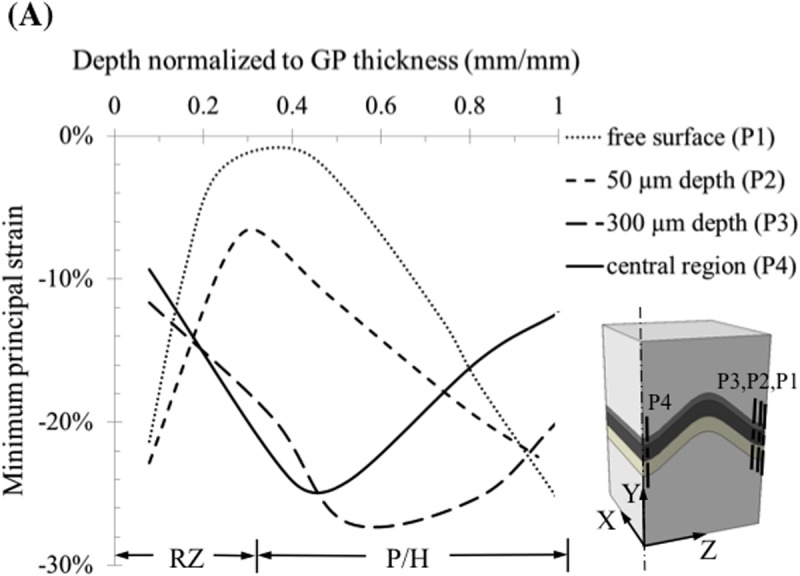
Distribution of minimum principal logarithmic strains within the ‘m’ shape growth plate obtained from the mesoscale model without cellular detail. Peak compressive strains occurred at the base of the proliferative zone where cell mitosis occurs. This contrasts with regions close to the free surface, where compressive strains were at a minimum around the base of the proliferative zone and maximum in the reserve and hypertrophic zones.

Principal strains were oriented along and transverse to the chondron directions throughout the central region but deviated from these directions at the reserve zone/bone interface near the free surfaces where shear strains developed along the bone interface (Fig 6). These results were obtained from the multi-scale model without chondrocytes. Planes 50 and 300 μm away from the free surface were chosen as being potentially relevant range for the plane of observation in microscopic studies of cell deformation under compression. The strain distributions were significantly different at these locations compared to interior regions of the growth plate. Minimum principal (compressive) strains at the tissue level varied throughout the growth plate zones. In the interior regions of the growth plate the minimum principal strains varied from -10% in the reserve zone peaked at -25% at the base of the proliferative zone and decreased to -10% in the hypertrophic zone (Fig 7). The opposite pattern was observed near the growth plate free surfaces. Similar trends were observed for both growth plate shapes (results not shown).

**Fig 8:**
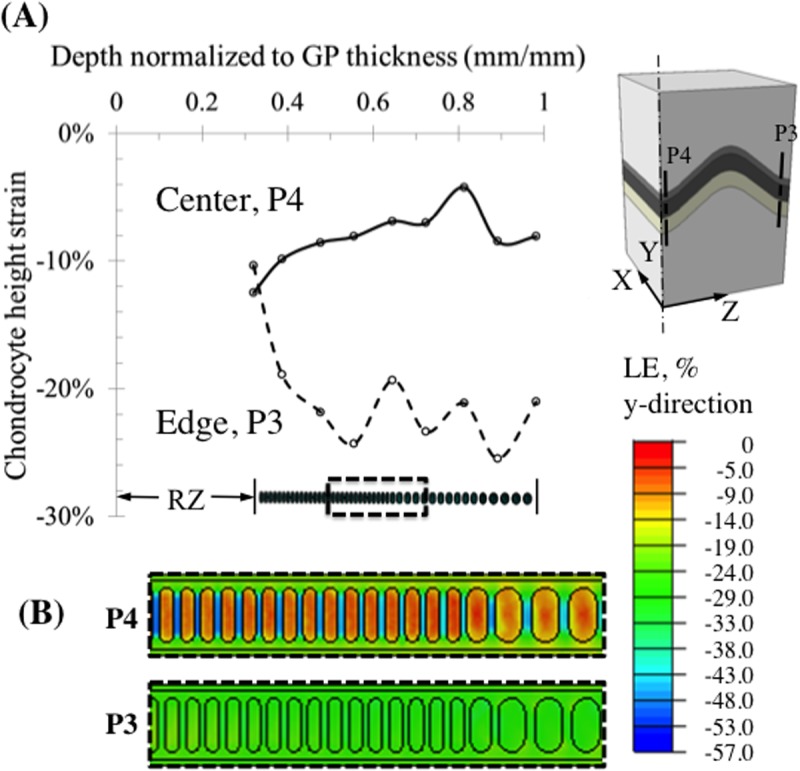
Chondrocyte deformation measured as change in cell height at two locations in the chondron for an overall axial compressive strain of 20%. (A) Cellular strains vary differently within each chondron depending on the location of the chondron. Within interior chondrons compressive axial cell strains peaked at the transition from reserve to proliferative zone where cell division occurs. Chondrons located close to the free surface (300 μm from the surface) of the growth plate experienced a reverse strain pattern with peak compressive strains occurring in the hypertrophic zone where no cell division occurs, but where cell size increases 10-fold. (B) Contour plot of the logarithmic strain (LE) at the chondron scale in the y-direction at two locations, at the center (P4) and at 300 μm from the edge (P3). The contour plot is obtained by extrapolating the integration point values to the nodes and then averaging.

**Fig 9:**
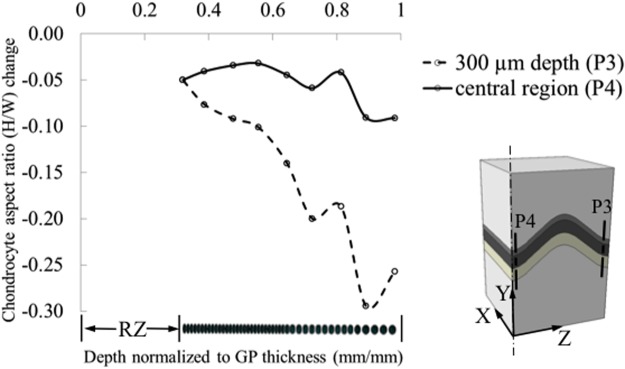
Chondrocyte aspect ratio change with deformation at the center and 300 μm away from the edge. Aspect ratio is defined as the ratio of the height over the width of the cells.

Chondrocytes in the interior of the growth plate experienced less strain (change in cell height) than the macroscopic tissue level strains with peak values of -12% in the center, along the P4 line, and -26% near the growth plate free boundaries (Fig 8). The variation in cellular height strain along the chondrons followed a similar trend to the zonal variation seen at the tissue level. When the chondron was located in the central region of the growth plate, the proliferative chondrocytes attained larger compressive strains (-12%) than hypertrophic chondrocytes (-6%). As was the case for tissue strains, the opposite pattern was seen for chondrons located near the free edges of the growth plate. The change from initial state in the aspect ratio (height/width) of the compressed chondrocytes displayed a similar pattern to that of the height strain. The cells in the hypertrophic zone near the free edge displayed the greatest change reaching a change in aspect ratio of approximately -0.3 (Fig 9).

## Discussion

In this study, a multi-scale computational approach was undertaken to better understand how physiological loads are experienced by chondrocytes embedded inside chondrons when the growth plate is subjected to displacements corresponding to moderate instantaneous compressive global strain. Specifically, we sought to determine if chondrons and the contained chondrocytes were exposed to similar deformations close to the cut surface as they do in the central region of the growth plate and to explore the relationship between computed strains and the cellular anatomy and physiology in different zones of the growth plate.

Our results indicate that chondrocytes in the interior of the growth plate undergo about 50% less strain than the prescribed overall growth plate strain. However, there is disagreement in the articular and growth plate cartilage literature regarding the macroscale strains whether they are reduced or amplified once transmitted to cellular scale. Some studies support a similar conclusion to ours that the cells experience lower strains than their surroundings [41]. Other studies show that chondrocytes are subjected to greater levels of strain than their surrounding tissue (strain amplification) [12, 42, 43] or that strain amplification depends on the level of tissue strain as well as the zonal location of the chondrocytes within the articular cartilage [44]. These discrepancies exist due to multiple factors, such as experimental methods employed, animal species, tissue type, etc. Cells in the most peripheral regions at the free boundaries of our model reached strains close to the prescribed overall growth plate strain.

It could be argued that assuming incompressibility of the cells, while allowing the cartilage tissue to be slightly compressible, could artificially engender lower cellular strains than the prescribed overall growth plate strain. To address these concerns we conducted additional studies in which we set Poisson’s ratio of the cartilage reserve zone, TM and ITM to be equal to 0.495, while keeping Poisson’s ratio of the cells at 0.50. Similar trends were obtained for this model with less compressible cartilage properties as for the original more compressible cartilage model (results not shown). Cells in the interior region experienced strain magnitudes of 50% or less than the prescribed overall growth plate strains in both cases. In the case of the more compressible cartilage model the peripheral cells attained strains in the range of the prescribed overall growth plate strain, but for the less compressible cartilage model peripheral cell strains ranged from slightly less to 60% more than the applied strain. Regardless of existing issues and discrepancies, this is an important question that needs to be considered and we present computational evidence suggesting that the growth plate cartilage cells inside chondrons in the interior of the growth plate experience less strain than the surrounding matrix when a moderate level of instantaneous strain is applied globally.

Our results demonstrate that the strain patterns along the length of the chondrons are reversed in peripheral regions of the growth plate in comparison with central locations, meaning that experimental measurements of tissue and cell strains may not be indicative of the true mechanical state that exists throughout most of the sample or to the *in vivo* mechanical state. These findings have implications for experimental studies where microscopic observations are made near the surface and for the design of bioreactors and artificial scaffolds developed for bone and cartilage tissue engineering

The results of our computational study may be compared to the few published experimental studies [12, 14, 45] of cell and tissue strains observed during growth plate compression. Since those studies involved measurements obtained from regions close to cut surfaces, the comparisons are limited to only those locations in the model (Table 2). Our model predicted that within 50 μm of the cut surfaces the reserve and hypertrophic zones experienced higher compressive strains (-20%) than the proliferative zone (-7% to -15%), which agrees with a previous study [14]. At the cellular level the model predicted that near the cut surface the chondrocytes in the hypertrophic and part of proliferative zone also reached slightly more compressive strains (-23%) than the applied macroscopic strain (20% of the unloaded growth plate thickness) in agreement with experimental observations [12, 45].

**Table 2:** Comparison of mean computed strains to reported mean experimental strains measured with confocal microscopy on compressed growth plate explants.

Study level	Applied strain (%)	Strain measure	RZ strain (%)	PZ strain (%)	HZ strain (%)	Epi- and meta-physeal bone left on explant	Thickness of RZ + P/H zones (mm)	RZ thickness (% of physeal height)	Study
tissue	-20	PC*- surface (50 μm depth)	-14	-14	-20	Yes	0.67	31	Present computational model
		PC*-interior	-10	-22	-14				
cell	-20	Surface (300 μm depth)	n/a	-20	-23				
		Interior	n/a	-9	-7				
tissue	-5	PC**	-9	-5	-9	Yes	about 1	< 10	Villemure et al. 2007 [14]
tissue	-10	Axial (long bone axis)	-2	-9	-4	No	3.4	70	Amini et al. 2013 [45]
		Transverse	+1	+1	+2				
cell	-15	Minor diameter	-8	-20	-18	No	3.2	70	Amini et al. 2010 [12]
		Major diameter	-11	-14	-18				

RZ: reserve zone. PZ: proliferative (columnar zone). HZ: hypertrophic zone. PC: principal compressive *logarithmic (present study) or **Lagrangian strain (Villemure *et al*.) strain. Surface: strains within certain depths of sample surface. Interior: strains at central region of the sample. Axial strain: along the bone long axis. Cell strains were reported as minor and major cell diameter changes. Minor diameter: cells in the PZ have minor diameters in line with the axial direction.

The *in vitro* mechanical response of the chondrons near the cut surface may be altered by multiple factors. The compressed ends of the samples may not have calcified cartilage or bone remnants as these boundary conditions were not clearly identified in the all of the published papers, but presumed to have been removed. The glass coverslip utilized in the imaging studies may have also prevented the bulging that we observed. Furthermore, our model was partially derived using a data-driven approach from experiments on fully developed growth plate explants at a stage following the completion of the secondary center of ossification rather than the early stage where the chondron-epiphysis is still predominately cartilaginous as appeared to be the case for two of the comparison studies [12, 45]. Younger growth plates that are thicker (3 mm) as were reported in two previous studies [12, 45] also had thicker reserve zones, which may affect the transverse outward bulging at the free surface differently.

Our current model assumes the cells behave as an incompressible elastic material and is therefore limited to short time, fast occurring loading where the fluid component has no time to move through the tissue. Under such instantaneous loading conditions, we assumed that the volume of the chondrocytes remained constant [40, 46]. We believe that the assumption of linearly elastic material behavior is reasonable for the purpose of describing the basic mechanical behavior of the growth plate under uniaxial compression [20, 25]. During compression of cells at short times scales (~0.5 s) intracellular water is redistributed within the cell [47], but this does not imply compressibility of the overall cell volume. At long time scales constant external pressure induces cell shrinkage, but cell shrinkage takes an order of magnitude (~10 s, [47]) longer than the transient impact of heel strike or even the 0.2 s duration of the stance phase of a gait cycle and as long as an hour to reach equilibrium [48]. However, since no short time scale cell strain measurements are available for comparison, we made comparisons to experimental studies conducted under equilibrium conditions and it is not known what effect time-dependence would have on the parameters that were compared.

Although not strictly relevant to our current short time-scale model for cyclical or transient loading conditions, we explored the influence of assuming slight compressibility (near incompressibility) of the cells on the calculated cellular strains. We obtained solutions for cellular Poisson’s ratios between 0.49 and 0.50 and found the solutions to be numerically stable and free from issues of mesh locking. However, these solutions did demonstrate that introducing near incompressibility into the elastic solution has large effects on the cellular strains, a finding which is consistent with the findings of others and therefore not one of numerical methods or issues related to numerical constraints [49]. It has been shown [49] mathematically that in problems involving shear deformations the normal stresses can exhibit extreme sensitivity to small changes in Poisson’s ratio that are especially acute for Poisson’s ratios between 0.495 and 0.50. Our results are consistent with these analytical findings. This is another reason why we do not believe the equilibrium state can be correctly modeled with a purely elastic approach since the precise value of cellular Poisson’s ratio is difficult to determine under conditions when incompressibility is no longer a reasonable assumption.

**Fig 10:**
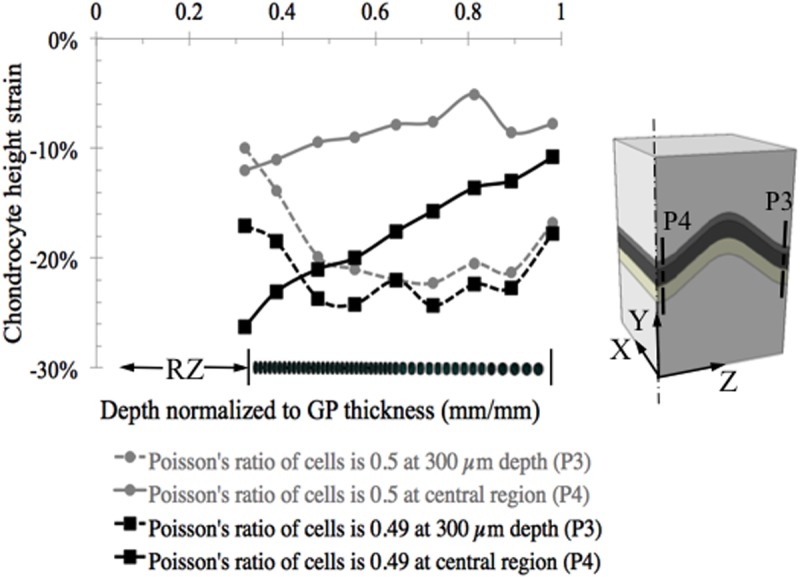
Results for two cases with the cellular Young’s modulus increased 20-fold over the instantaneous short time scale value, for cellular Poisson’s ratio = 0.50 and 0.49 in an effort to approximate long-time scale compression with a purely elastic approach to compare with the experimental values in the literature. Results are very sensitive to assumed values of cellular Poisson’s ratio and to the Young’s modulus ratio between the cell and the tightly bound territorial matrix and we do not believe this is the correct approach for modeling long-time scale events since the real value of cellular Poisson’s ratio cannot be accurately determined.

Nevertheless, if for the sake of comparison one assumes slight compressibility of the cells under equilibrium conditions, following cell shrinkage after prolonged compression (Poisson’s ratio = 0.49), it is also necessary to consider the fact that the Young’s modulus of the cell increases as the volume decreases [47], varying exponentially with the volume fraction of the remaining solid phase of the cell for long time scale events [50]. To simulate such a condition we compare two solutions (Fig 10) with the cellular Young’s modulus increased 20-fold over the instantaneous value, consistent with the increases measured experimentally [50]. The cellular strains were significantly changed under conditions of slight compressibility relative to the incompressible case, which was solved for the same 20-fold greater Young’s modulus in order to exclude the possible influence of changing the modulus. The cellular strains were more sensitive to small changes in compressibility for chondrons located in the central region than for the regions near the free surface or plane of microscopic observation.

As a partial exploration of the degree of sensitivity of the cellular strain results to assumed values for the material properties we decreased the value of Young’s modulus in the reserve zone, a major component of the growth plate, from 0.98 MPa to 0.42 MPa, an almost 60% decrease. Every other parameter was kept the same as in the original analysis (Fig 8). This resulted in a relative change in average chondrocyte height strain in the central region of 7.44% and in the peripheral region of 9.3%. We may therefore assume that the results are not very sensitive to the assumed values at the mesoscale (see S1 Fig). At the microscale, the region of the model most likely to influence the calculated cellular strains is the ratio of moduli for the cell and surrounding territorial matrix. As has been shown using transmission electron microscopy (TEM) the chondrocytes are tightly bound to the territorial matrix (TM) through an intervening thin layer of extracellular matrix, which we have not included in the model [51]. The TM in our model was kept constant along the entire chondron and it acts as a cushion absorbing the strains in the regions between the incompressible cells.

The application of epiphyseal displacement equivalent to 20% of the initial growth plate thickness was motivated by the need to obtain mesoscale values for Young’s modulus by simulating compression experiments on fully developed growth plate explants [37]. This prescribed displacement resulted in a 14.8% compressive growth plate strain across the combined layers of the reserve, proliferative and hypertrophic zones, which is representative of published explant growth plate experiments used for comparison (Table 2) and other growth plate explant experiments [38, 52–54]. Finite element simulations of rabbit experiments suggested that growth plate stresses reached values of 0.1 to 0.6 MPa in compression during activities such as sitting [18]. From the stress relaxation experiments [37] on explants it may be estimated that 0.2 MPa corresponds to the stress levels attained after stress relaxation for displacements of the epiphysis equal to 20% of the initial growth plate thickness (defined as the combined thicknesses of the reserve, proliferative and hypertrophic zones). We therefore assume that our model simulations are representative of physiological levels of strain.

Limitations to the model include simplification of the internal topography of the growth plate cartilage layer by employing two simplified shapes representing idealized shapes seen in growth plate experimental samples obtained from cow growth plates [34, 37]. Chondrocytes were assumed to be attached to the surrounding matrix in a continuous manner. Although the connections are believed to be discrete [55], modeling the chondrocyte/matrix interface as continuous appears to be an accepted practice [22, 56].

As a further limitation, since the macroscale models were built based on typical mechanical test samples and not on whole growth plate anatomy of the long bones, some structures were not included, such as the perichondrium, groove of Ranvier and ring of LaCroix. It is possible that these unique structures would provide some support to the growth plate cartilage and would lead to less distortion of chondrons as well as embedded chondrocytes located away from the central region of the bones. Since neither our models nor most previous experiments included the perichondrium, the true effects of edge effect from the free surface could be diminished with the existence of this structure. Therefore, it seems likely that observations from or close to the free surface during *in vitro* experiments on explants of growth plate cartilage cannot fully represent the *in vivo* situation.

**Fig 11:**
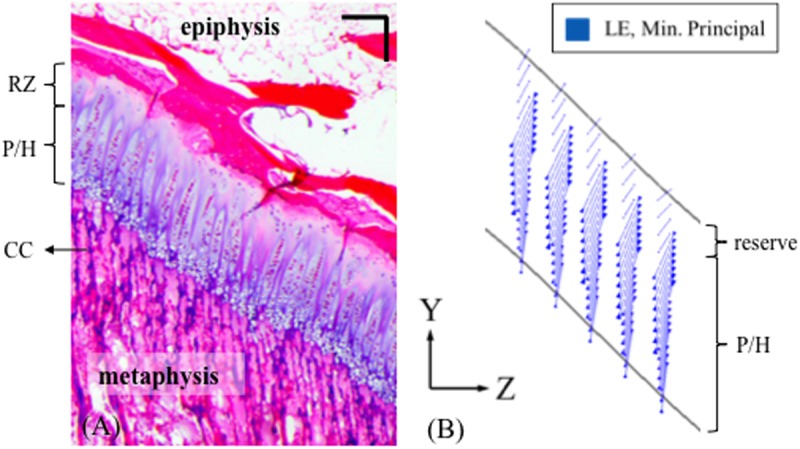
Chondrons appear to be oriented along the minimum principal strain directions, becoming roughly perpendicular to the epiphyseal bone plate, whereas the hypertrophic portion along with the calcified cartilage bars and primary spongiosa align more with the primary compressive load direction along the tibial long axis (Y). **(A)** Histology slice of a 4-month old bovine proximal tibial growth plate stained with H&E, scale bar = 200 μm. **(B)** Minimum principal strain vector plot at the central region of the ‘m’ shape growth plate. The results were obtained from the mesoscale model without the cellular detail.

An interesting observation in this study was that the orientation of chondrons of the growth plate followed the direction of minimum principal strains (Fig 11). It should be noted that this was under conditions of uniaxial compression along the Y-axis in the mesoscale model that had no chondron structures. Although this was obtained for instantaneous loading, this finding suggests that the direction of principal strains may influence bone growth development by directing the bone to grow along the principal compression directions.

In summary, this work provides a new approach to study growth plate behavior under compression and illustrates the need for combining computational and experimental methods to better understand the chondrocyte mechanics in the growth plate cartilage. While the current model is relevant to fast dynamic events, such as heel strike in walking, we believe this approach provides new insight into the mechanical factors that regulate bone growth at the cell level and establishes a basis for developing models to help interpret experimental results at varying time scales.

## Supporting Information

S1 Fig Chondrocyte deformation measured as change in cell height in the chondron at two growth plate locations (central and peripheral) for a prescribed displacement corresponding to an overall axial sample compressive strain of 20%. To explore the sensitivity of the cellular strain results to assumed values for the material properties of the reserve zone, a major component of the growth plate, we decreased the reserve zone Young's modulus from 0.98 MPa to 0.42 MPa, an almost 60% decrease. Every other parameter was kept the same as in the original analysis the results of which are shown (circles) along with those for the reduced modulus (squares). This resulted in a relative change in average chondrocyte height strain in the central region (P4) of 7.44% and in the peripheral region (P3) of 9.3%. We may therefore assume that the results are not very sensitive to the assumed values at the mesoscale.
(PDF)
