# Supplementary material for: Regional Variations in Growth Plate Chondrocyte Deformation as Predicted By Three-Dimensional Multi-Scale Simulations
Source: PLoS One. 2015 Apr 17;10(4):e0124862. doi: 10.1371/journal.pone.0124862 (PMC4401775; doi:10.1371/journal.pone.0124862)
Supplement: S1 Fig — To explore the sensitivity of the cellular strain results to assumed values for the material properties of the reserve zone, a major component of the growth plate, we decreased the reserve zone Young's modulus from 0.98 MPa to 0.42 MPa, an almost 60% decrease. Every other parameter was kept the same as in the original analysis the results of which are shown (circles) along with those for the reduced modulus (squares). This resulted in a relative change in average chondrocyte height strain in the central region (P4) of 7.44% and in the peripheral region (P3) of 9.3%. We may therefore assume that the results are not very sensitive to the assumed values at the mesoscale. (PDF) [file pone.0124862.s001.pdf]

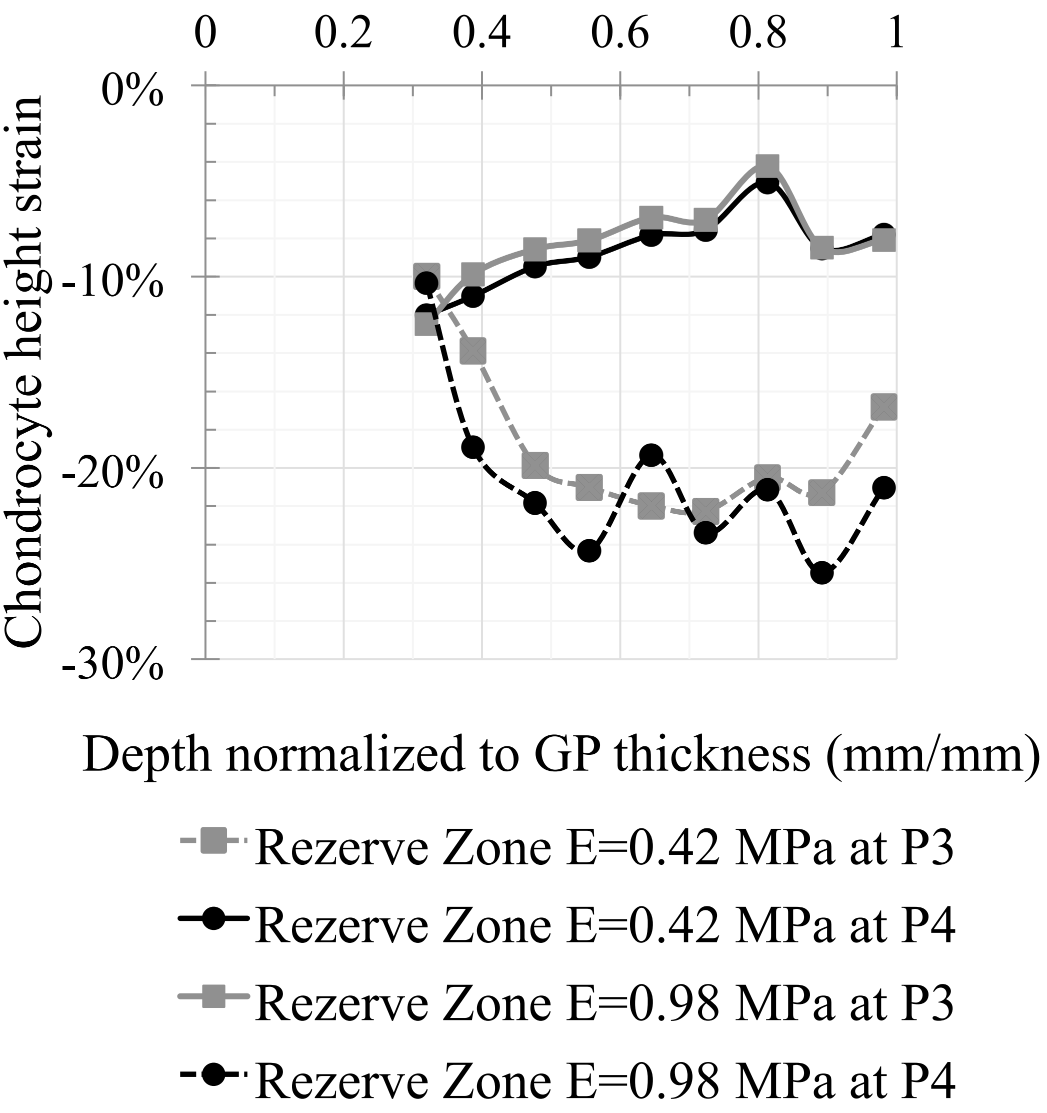
0
0.2
0.4
0.6
0.8
1
0%
-10%
-20%
-30%
Chondrocyte height strain
Depth normalized to GP thickness (mm/mm)
Rezerve Zone E=0.42 MPa at P3
Rezerve Zone E=0.42 MPa at P4
Rezerve Zone E=0.98 MPa at P3
Rezerve Zone E=0.98 MPa at P4
